# Extracting viscoelastic material parameters using an atomic force microscope and static force spectroscopy

**DOI:** 10.3762/bjnano.11.77

**Published:** 2020-06-16

**Authors:** Cameron H Parvini, M A S R Saadi, Santiago D Solares

**Affiliations:** 1 Department of Mechanical and Aerospace Engineering, The George Washington University School of Engineering and Applied Science, 800 22nd St. NW, Suite 3000, Washington, DC 20052, United States

**Keywords:** atomic force microscopy (AFM), creep, force mapping, indentation, Kelvin–Voigt, static force spectroscopy (SFS), viscoelasticity

## Abstract

Atomic force microscopy (AFM) techniques have provided and continue to provide increasingly important insights into surface morphology, mechanics, and other critical material characteristics at the nanoscale. One attractive implementation involves extracting meaningful material properties, which demands physically accurate models specifically designed for AFM experimentation and simulation. The AFM community has pursued the precise quantification and extraction of rate-dependent material properties, in particular, for a significant period of time, attempting to describe the standard viscoelastic response of materials. AFM static force spectroscopy (SFS) is one approach commonly used in pursuit of this goal. It is capable of acquiring rich temporal insight into the behavior of a sample. During AFM-SFS experiments the cantilever base approaches samples with a nearly constant velocity, which is manipulated to investigate different timescales of the mechanical response. This manuscript seeks to build upon our previous work and presents an approach to extracting useful linear viscoelastic information from AFM-SFS experiments. In addition, the basis for selecting and restricting the model parameters for fitting is discussed from the perspective of applying this technique on a practical level. This work begins with a guided discussion that develops a fit function from fundamental laws, continues with conditioning a raw SFS experimental dataset, and concludes with the fit and prediction of viscoelastic response parameters such as storage modulus, loss modulus, loss angle, and compliance. These steps constitute a complete guide to leveraging AFM-SFS data to estimate key material parameters, with a series of detailed insights into both the methodology and supporting analytical choices.

## Introduction

Modern AFM applications commonly involve testing samples that are soft, biological, or polymeric in nature. Understanding the dissipative nature of these materials at the nanoscale is especially important to their use in many applications [1–6]. However, performing such measurements using AFM has been difficult due to the complexity of material phenomena that govern AFM observables. For example, the history- and rate-dependence of tip–sample interactions can produce large errors when estimating the surface stiffness, leading to poor and inconsistent data quality. To avoid complex analytical derivations, it can be convenient to ignore well-established viscoelastic models in favor of elastic relationships. Unfortunately, these equations are sometimes oversimplified to such an extent that they no longer properly represent the behavior of real materials [7–8]. Such approaches critically lack the capability of reproducing the complex temporal behavior of samples with one or more characteristic times. It follows that more detailed viscoelastic models are necessary to describe and predict AFM tip–sample behavior accurately [9–12]. To this end, an appropriate viscoelastic model must be chosen (or derived) and subsequently specified for a geometry specific to AFM tip–sample interactions.

The process of developing and fitting a viscoelastic model is somewhat organic. One approach is to use theoretical arguments in conjunction with continuum mechanics fundamentals, but it is most common to design more intuitive spring–dashpot linear viscoelastic mechanical models [13–15]. The spring–dashpot, or “mechanical equivalent” approach to linear viscoelasticity involves connecting different combinations of springs and dashpots in series and/or parallel to mimic the action of a material. These physical systems are then described algebraically, transformed into Laplace space, and rearranged to create transfer functions that describe the material response for a given stress or strain excitation. The mechanical-equivalent approach is simple to explain, but can require more assumptions and some additional knowledge of the Laplace transform to derive analytical stress–strain relationships. Alternatively, continuum mechanics can be used to create more general models to describe the material under study. The continuum method involves the derivation and restriction of constitutive relations that apply to practical problems in mechanics. These relations are then subjected to balance laws in order to develop a pipeline from problem statement to final configuration [16]. The continuum approach is more general at the cost of requiring significant expertise and complex explanations that are more difficult to follow.

Either method is capable of generating a model that provides significantly more insight into the material response than elastic models, and the choice between approaches usually depends upon the material and complexity of interactions being represented. In the case of AFM, many common problems involve nanoscale viscoelastic systems exposed either to intermittent probe contact (tapping mode) or a similar type of probe–sample interaction where the indentations are usually small and fast. In such cases mechanical-equivalent models usually suffice, and the continuum approach is more complicated than necessary to reproduce the material response. When the contact occurs for longer periods, such as during contact-mode AFM or when tip–sample nonlinearities are clearly visible in the dataset, continuum models may be necessary to capture all of the deformation complexity. Using either technique, the goal remains to create a physically accurate model of the sample, and use experimental data to quantify meaningful material properties.

Lopez et al. [17] recently formulated a generalized solution to the physical contact problem in AFM-SFS experiments, particularly for cases where the sample is viscoelastic. The approach is also capable of being adapted to any linear viscoelastic stress–strain model. Their method utilized a theoretical basis for viscoelastic indentation problems proposed by Lee and Radok [18] in 1960. Lee and Radok presented a framework that used Hertzian contact relationships and the “viscoelastic correspondence principle”, in which elastic parameters are replaced by their viscoelastic counterparts to account for differences in the mechanical response of the material. Approaches utilizing viscoelastic correspondence were then cited frequently in the literature of that time [19–21].

The aforementioned work of Lopez et al. focused upon materials with an arbitrary number of retardation times and included analytical descriptions both with and without the assumption of a linear force-vs-time curve. This generalized approach neatly ties together the theory of Lee and Radok with a modern understanding of linear viscoelastic mechanical-equivalent (i.e., spring–dashpot) models, and the present article seeks to provide a more detailed discussion on practical applications of the methodology of Lopez and co-workers. Topics discussed here include the basis in theory, how to specify various material models for fitting, how to calculate relevant viscoelastic quantities, and importantly a discussion of where the extracted parameters are valid. The extension of this methodology to nonlinear viscoelastic materials is the subject of a future manuscript in development. In order to begin extracting viscoelastic parameters, the solution outlined by Lee and Radok [18] must be revisited and subsequently specified for an AFM-SFS experiment as described by Lopez and co-workers [17]. Afterwards, the relationships are generalized for an arbitrary load history and assumptions are made about the fundamental characteristics of the material being studied. The “Theoretical Background” section focuses on the analytical approach, with the detailed data handling outlined in the section immediately afterward. For clarity, a specific example is then provided in the “Results and Discussion” section, showing how to extract viscoelastic parameters using AFM-SFS data collected from hollow nylon 6,6 tubing.

## Theoretical Background

To understand the analytical choices available for viscoelastic models in AFM indentation problems, it is helpful to begin with the viscoelastic correspondence principle framework mentioned above. An AFM tip–sample interaction involves measuring force and deformation, which can then be compared to the solution of a contact problem where a spherical probe tip indents a flat viscoelastic surface. Depending upon the material being tested, different models can be inserted into the resulting force–indentation relationship and then compared to AFM-SFS data. The parameter values within the model are modified until a close approximation is made between the experimental force and indentation datasets, resulting in a set of “best-fit” viscoelastic parameters for the model. The following sections explain this workflow in a similar manner: First, the framework is described briefly, then the force–indentation relationship is modified to be more easily compared with AFM data, and last, the features of several viscoelastic models are discussed. For a detailed derivation of the Lee and Radok framework, and a review of the extension to arbitrary load history (previously presented by Lopez et al. [17]), the reader is directed to Supporting Information File 1 included with this manuscript.

### Spherical indentation of a viscoelastic half-space

**Figure 1:**
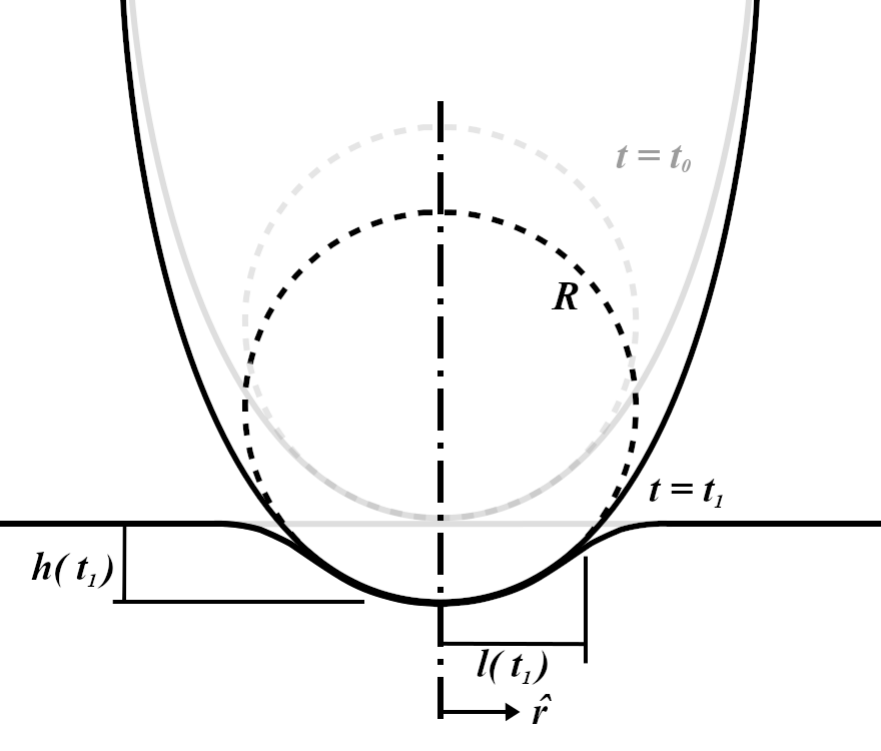
The quasi-static spherical indentation configuration as outlined by Lee and Radok in [18].

In an ideal case, the exact tip geometry is known a priori. In lieu of such information, it is expedient to make simplifying assumptions about the tip shape at the point of contact. One common assumption is that the local surface deformations are small, such that the tip has a roughly spherical contact geometry during indentation. This assumption is best made for larger-diameter tips or specialized colloidal probes, although it can be argued that smaller tips can still be approximated provided the indentation depth is small. As mentioned above, Lee and Radok [18] proposed a solution to the rigid spherical contact of a viscoelastic half-space nearly sixty years ago. The indentation configuration is visualized in Figure 1, which is based upon Figure 1 in their original manuscript.

Here, the deepest indentation occurs at the center of the sphere (*r* = 0) and is labeled *h*(*t*). The indenter has a radius of curvature *R*, and the distance from the center axis to the edge of contact, known as the contact radius, is labeled *l*(*t*).

Using the geometry presented in Figure 1, a series of algebraic manipulations, Laplace transformations, and viscoelastic-equivalence arguments, Lee and Radok presented the following relationship between applied load and spherical indentation depth for a viscoelastic material having characteristic operators *u* and *q*

[1]
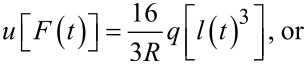


[2]
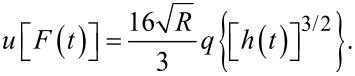


Equation 2 is the relationship upon which Lopez et al. build a solution for the AFM experiment. It is originally presented as Equation 3 in their paper [17].

### Extending the solution to an arbitrary load history

Traditionally, when using creep-recovery experiments to parameterize the viscoelastic models under study, a constant stress is first applied to a sample and then subsequently removed. For example, one method involves hanging weights from a material for some time, then removing the weights and allowing the material to recover. A strain gage or displacement sensor captures the deformation occurring during both phases, and the data is used for fitting. The boundary conditions for such an approach dictate a step function in stress, and that one end of the sample is fixed. In that case, the constant load history would suggest using the “creep compliance” *J*(*t*), an engineering quantity that represents the change in strain as a function of the time for a medium exposed to an instantaneous constant stress [17–18]. While applicable for this type of study, the tip–sample interaction during AFM experiments does not apply a constant force (i.e., stress) as a function of the time. The load history is more reminiscent of a discrete impulse function in which the contact time is short. Therefore, while the solution form could use the creep compliance for fitting, it is more direct to use the material retardance. The creep compliance can be defined in terms of the applied stress σ(*t*), the strain ε(*t*), and the material retardance 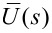 as:

[3]
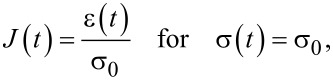


[4]
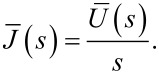


By taking the Laplace transform of Equation 2, rearranging, substituting the creep compliance relationship above, and taking the inverse Laplace transform, one arrives at the following result:

[5]
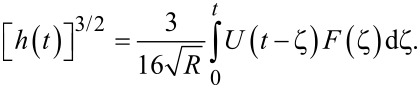


Equation 5 allows for the straightforward definition of the retardance *U*(*t*) according to an appropriate material model. The convolution integral replaces the multiplication of 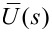 and 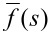 in the Laplace domain during translation back to the time domain. As mentioned previously, the approach outlined here is preferred for AFM as it both removes the requirement of a step function in stress and of a measurement of the force application rate. Since an AFM-SFS experiment provides force and indentation (deformation), the data streams can be utilized directly without requiring a discrete derivative of force in time. Taking a discrete derivative can introduce undesirable oscillatory errors into the resulting dataset, and thus obscure some of the information contained within. Equation 5 represents the strain (or deformation) response of a material to the unit stress impulse.

### Specifying a viscoelastic material model for fitting

Selecting an appropriate material model is often an iterative process, unless one can leverage available datasets or a priori knowledge about the nature of a given sample. There is a broad array of literature that covers spring–dashpot models and viscoelastic rheology, many of which provide helpful visualizations of the predicted material response [14–15,22]. Provided a relationship between model parameters and the material retardance can be derived, one can replace *U*(*t* − ζ) in the above equations to make an implicit assumption of how the material reacts to applied stress. Acquiring these relationships is generally done by taking the derivative of the creep compliance *J*(*t*) of the model, as the two quantities are related in Laplace space by Equation 4. Table 1 summarizes several commonly used viscoelastic models and their reported applicability.

**Table 1:** Summary of viscoelastic models and the corresponding applications [14–15,22].

type	name	shape	stress–strain equation	parameters

linear	Kelvin–Voigt^a^	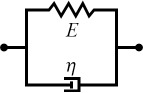	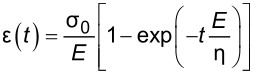	2

	standard linear solid (Voigt form)^b^	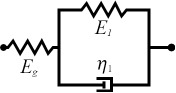	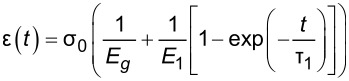	3

	generalized Kelvin–Voigt^c^	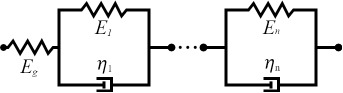	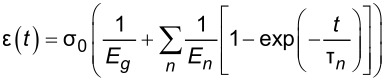	1 + 2*n*

	Maxwell^d^	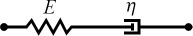	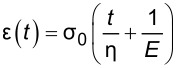	2

	Wiechert^e^	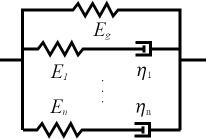	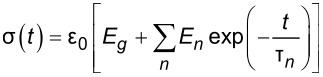	1 + 2*n*

nonlinear	Schapery^f^	—	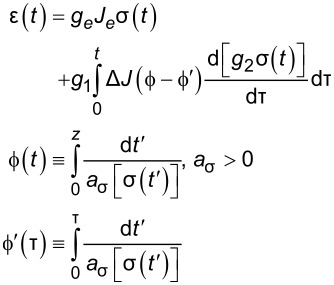	7+

^a^Model includes exponential (and reversible) strain creep under stress, but no stress relaxation with constant strain. The response will decay to zero residual stress provided sufficient time. ^b^Model includes both creep and relaxation phenomena, but is not accurate when literally compared to common material responses. ^c^If including the fluidity term, an additional dashpot is added in series with the mechanical model. This model accounts for creep strain occurring at multiple retardation times. ^d^Model includes exponential (and reversible) stress relaxation under constant strain, but linear (non-reversible) strain creep under constant stress. ^e^Most easily applied to relaxation experiments (constant strain). This model accounts for relaxation occuring at multiple time scales. ^f^By including additional terms in ϕ and ϕ^′^, more environmental effects can be accounted for and the number of parameters increases.

Often these linear spring–dashpot descriptions utilize an expanded series of compliances and characteristic time constants, which introduces a common source of error during analysis of the viscoelastic data. Each term in the series is intended to represent the reaction of the material to applied stress on a particular time scale, represented by τ in the governing equations shown in Table 1. As stated previously, provided the model has a derived retardance, it can be inserted into Equation 5 directly without an inherent restriction on the number of characteristic terms included in the series. Often additional terms are inserted with the intent of more accurately fitting the dataset in question. However, if the data does not include information at those timescales, whether due to a lack of resolution (for small time scales) or limitations on the experiment length (long time scales) these additional terms will introduce significant error in the parameters extracted. Furthermore if the parameter set obtained is extrapolated for new loading conditions, it will incorrectly predict the material response as a result of an incorrect number of terms (i.e., branches in the generalized mechanical-equivalent model).

It is critical to evaluate the timescales contained within a dataset before performing a parameter extraction, especially, before predicting new material responses. For example, since modern AFM instruments are capable of acquiring data on the scale of 2 kHz for quasi-static characterization, the smallest characteristic timescales that can be resolved are of the order of 10^−4^ s. If parameters are fit to a dataset of the order of 10^−4^ to 10^0^ s (e.g., data collected at 2 kHz for a total experiment length of 1–3 s), it is improper to utilize that parameter set to mimic the action of a material for an experiment lasting more than 10 s. Issues of this kind can lead to significant problems with the performance of a parameterized model. As technology continues to advance and AFM setups can sustain faster data acquisition speeds, the resulting datasets will continue to become richer in time-dependent material information and provide a more complete understanding of how the sample material responds to stress.

To maintain consistency between the approach outlined here and that of Lopez et al., the generalized Kelvin–Voigt model has been selected for analysis. The retardance is usually found by taking the derivative of the compliance of a model [17]:

[6]
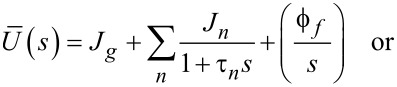


[7]
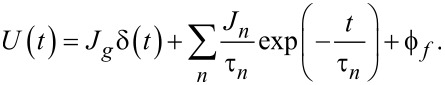


Several new parameters have been introduced. The first is the “glassy compliance” (*J_g_*), representing the elastic response of the material at short timescales; the second is the characteristic time (τ*_n_*), which scales the third parameter, the characteristic compliance (*J_n_*), such that it contracts on a specific timescale corresponding to the *n*-th branch of the generalized Voigt model. By inserting Equation 7 into Equation 5, we obtain:

[8]
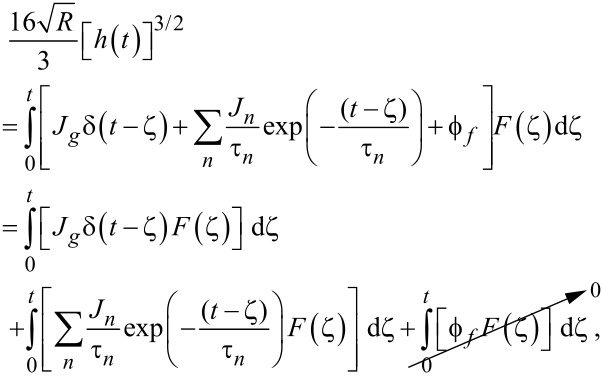


[9]
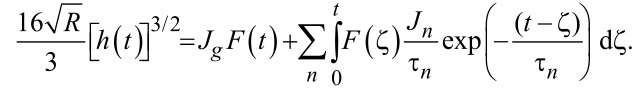


Note that the final term containing the steady-state flow ϕ*_f_* has been removed as a higher-order effect with relatively small magnitude for mostly solid viscoelastic materials. Materials that exhibit steady-state flow under stress, such as plastic or glass at high temperatures, are thus excluded from the analysis shown here. If this term was included under special circumstances, the set of fit parameters would increase by a number determined by the steady-state flow model utilized for ϕ*_f_* and an additional numerical convolution would become necessary.

Equation 9 allows for extracting parameters for *n* characteristic terms without any assumptions about the load history, provided the penetration grows monotonically, the indentation is not “sufficiently large”, and the correspondence principle applies. It represents the objective fit function described by Lopez et al. [17] and requires a numerical convolution of *U*(*t*) and *F*(*t*) in terms of the material parameter set {*J_g_*, *J_n_*, τ*_n_*}.

### Useful viscoelastic quantities

When characterizing the response of viscoelastic materials to external stress, especially during cyclic loading (such as with dynamic mechanical analysis (DMA) machines), it is common to calculate harmonic quantities such as the storage modulus *E*′, loss modulus *E*′′, and loss angle δ [23–25]. Each has a physical interpretation, representing the elastic and viscous motion of the material and their magnitudes relative to one another. For example, a material that is very stiff will have a high storage modulus and a low loss modulus. Such a sample will tend to store a majority of the applied load within its molecular structure and elastically return most or all of that energy when unloaded. Alternately, a medium that is susceptible to large shear forces (such as fluids) will have a low storage modulus and high loss modulus. In this case, most of that input energy will be lost to friction and heat, and therefore the material will return far less energy than the stiff elastic material when unloaded. To acquire storage and loss modulus as functions of the frequency, a Fourier transform of the compliance equation is applied to a particular model. Once translated into the frequency domain, the real portion of the expression corresponds to the storage modulus, and the imaginary portion to the loss modulus. In the case of a generalized-Voigt material, a definition in terms of modulus is analytically inconvenient. The model is most clearly written using material compliances (*J_n_*), and as such the storage compliance and the loss compliance (*J*′ and *J*′′, respectively) are used. The corresponding moduli can be written as [26]:

[10]
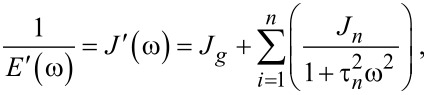


[11]
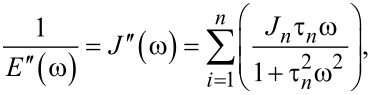


[12]
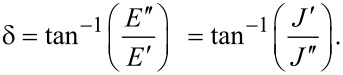


For ideally elastic behavior, the storage modulus or compliance dominates and the loss angle tends towards zero. In contrast, when the dissipative capacity of the material dominates, the sample action is more fluid-like and the loss angle increases. Understanding these viscoelastic quantities in the context of material characterization can be helpful when describing the relationship between external loads and the viscoelastic response, especially when the excitations are periodic. Within the context of AFM, both the storage modulus and the loss modulus are critical to evaluating the dissipated energy during tapping-mode analysis [24]. The key equations required for viscoelastic parameter inversion are as follows:

Equation 2, general Lee and Radok solution for spherical indentation [18]:


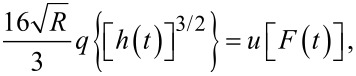


Equation 5, integral form of Lee and Radok spherical indentation solution [17]:


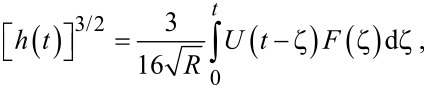


Equation 9, Lopez solution for spherical indentation of generalized-Voigt materials with multiple retardation times [17]:


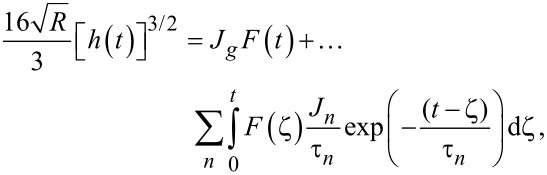


Equation 10, storage compliance for generalized-Voigt viscoelastic materials [26]:


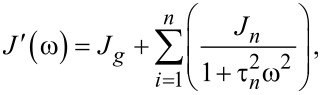


Equation 11, loss compliance for generalized-Voigt viscoelastic materials [26]:


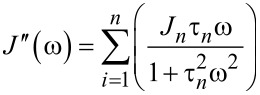


Equation 12, loss angle for generalized-Voigt viscoelastic materials [26]:


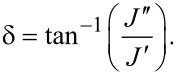


## Results and Discussion

### Extracting viscoelastic parameters

The following sections discuss how to leverage AFM-SFS data for viscoelastic model parameterization, including how to condition raw datasets for fitting, extract material model parameters, and calculate the viscoelastic quantities mentioned above. While the current approach is primarily geared towards MATLAB implementation, the original process was outlined by Lopez et al. [17] in Python, and is available in a public Github repository.

#### Conditioning raw static force spectroscopy datasets

**Figure 2:**
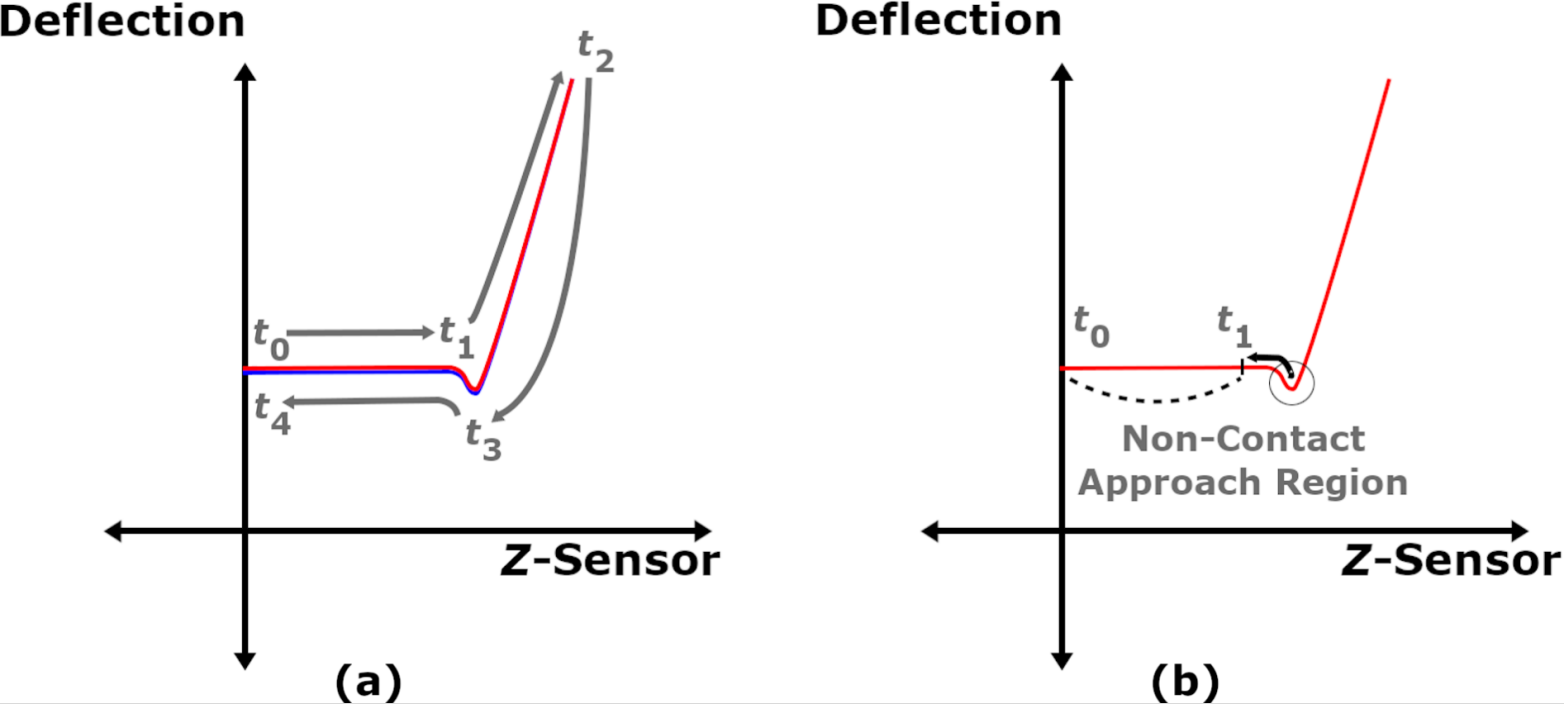
(a) A raw AFM-SFS dataset; (b) approach portion from the raw curve.

Traditionally, AFM-SFS experiments generate a variety of data streams for post-processing, including *Z*-Raw (expected *Z*-position based on the voltage applied to the *Z*-piezo), *Z*-Sensor (measured *Z*-position), deflection, amplitude, and phase. The *Z*-Sensor measurement corresponds to the position of the piezo that controls the position of the base of the cantilever, and the time-derivative of the *Z*-Sensor data should be equal to the experimental approach velocity. To perform the fit procedure, only the deflection and *Z*-Sensor datasets are necessary. Figure 2a illustrates the shape of a common result as read from a force–distance curve output file.

Because the Lee and Radok derivation used previously is restricted to a monotonically increasing surface area, the approach portion of the dataset must be isolated for analysis. The maximum *Z*-Sensor point marks the beginning of retraction, and therefore all points occurring after the time where that reading occurs (*t*_max(_*_z_*_)_, marked as *t*_2_ in Figure 2a) are removed from the dataset. The resulting curve is illustrated in Figure 2b.

**Figure 3:**
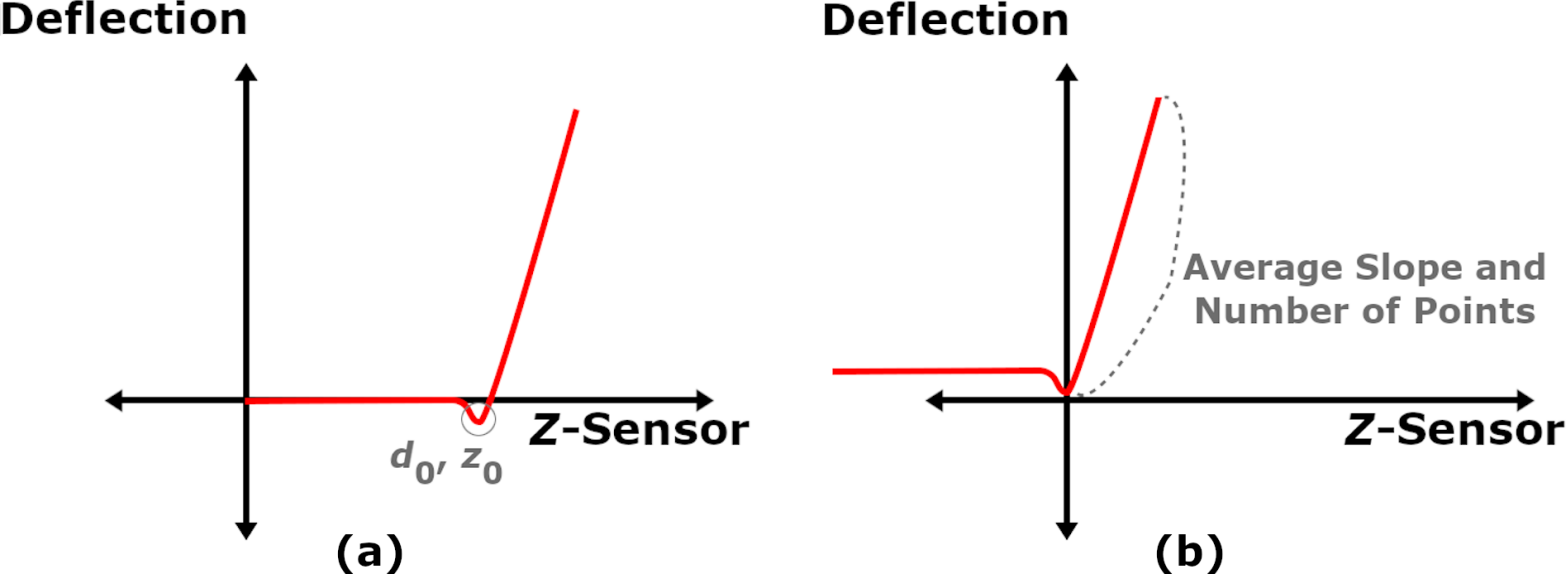
(a) AFM-SFS data corrected for an initial deflection offset; (b) Correction of the *Z*-Sensor dataset such that the minimum cantilever position occurs at the origin.

Due to a number of possible factors, e.g., calibration and noise, the deflection in the region *t*_0_ to *t*_1_ can be non-zero despite the probe not being in contact with the surface. To correct for this error, one needs to first locate the minimum deflection point. From there, the user needs to determine a sufficient step backwards in time such that the probe has yet to experience both long-range attractive forces and “snap-in”. The resulting time is marked as *t*_1_, and the average deflection offset is calculated using the deflection data from *t*_0_ to *t*_1_ (recall that the deflection should be set to zero at *t*_0_). The offset is then subtracted to produce the corrected deflection (*d_c_*). The dataset should now show the deflection as roughly zero for the period before snap-in, as seen in Figure 3a.

It remains to adjust the dataset such that the point of minimum deflection during snap-in coincides with the origin of the deflection-vs-*Z*-Sensor curve. This step simplifies calculating the slope of the curve where the force is increasing as a function of the time. While not explicitly necessary, smoothing the deflection data with a simple first-order Butterworth filter can remove some of the measurement noise and make finding the deflection minimum easier. While searching for the smallest value without filtering, noise can cause neighboring points to appear lower than the actual minimum and introduce error. It is convenient at this stage to store both the *Z*-Sensor and the deflection values at the time of minimum deflection as *z*_0_ and *d*_0_. The corrected *Z*-Sensor values (*z_c_*) are then calculated according to:

[13]
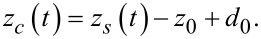


The applied tip force (*F*(*t*)) and indentation depth (*h*(*t*)) vectors can then be calculated using the cantilever stiffness (*k_c_*), the minimum deflection offsets (*z*_0_, *d*_0_), the deflection (*d*(*t*)), and the following relations:

[14]
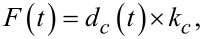


[15]
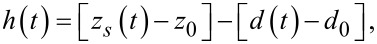


where *k_c_* in Equation 14 is the AFM cantilever stiffness. This data is not used directly in the fit, but can be useful for showing the indentation and force during the approach of the tip into the sample. The final conditioning step requires extracting the repulsive portion from both of the corrected datasets (*z_c_*(*t*) and *d_c_*(*t*)). While there are a variety of methods for identifying where the repulsive data starts, an easily implemented method involves creating a linear approximation of the repulsive *Z*-Sensor data *z*_rep_. The slope 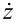 is taken from the corrected *Z*-Sensor data within the region noted in Figure 3b, which begins at the time where the deflection minimum occurs (*t*_min(_*_d_*_)_), and the number of points *n_p_* in that region is counted. Generating the *z*_rep_ vector is then performed by creating an array from 0 to *n_p_* in steps of 1 and multiplying by 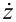. Subtracting the repulsive potion of the deflection (*d*_rep_) from *z*_rep_ provides the tip position relative to its neutral point:

[16] $$d_{\text{rep}}\left(t\right)=d_{c}\left(t>t_{\text{min}(d)}\right)-d_{c}\left(t_{\text{min}(d)}\right),$$

[17] $$z_{\text{tip}}\left(t\right)=z_{\text{rep}}\left(t\right)-d_{\text{rep}}\left(t\right).$$

The data utilized for fitting is the region where *z*_tip_ is greater than zero, indicating the tip has begun penetrating the surface and coinciding with the start of force application on the sample. Trimming data before this point provides the final form of the time array *t*, the repulsive deflection *d*_rep_, and the repulsive *Z*-Sensor values *z*_rep_ utilized in the following sections. In the following, the subscript “rep” will refer to these final forms where the force application has begun. To conclude the data conditioning, Equation 14 and Equation 15 are used again to calculate the experimental force *F* and the indentation depth *h* with repulsive data. At this point it is recommended to save re-sampled, log-scale versions of both *F* and *h* for use during the fit process. Because Equation 9 will be parameterized using a nonlinear least-squares approach, the number of data points per decade will drive the fit quality on that timescale. Ensuring that there are an equal number of data points in each log order will lead to consistent weighting across the entire experimental time scale [17].

The conditioning thus far has focused on preparing a single AFM-SFS dataset. However, most experiments involve multiple runs at a single site, which is then repeated at multiple locations on the sample to acquire a broader description of the surface properties. The mechanical responses can be averaged to both reduce computational overhead during fitting and give a prediction that is less sensitive to inhomogeneities. However, care must be taken to avoid issues that can arise when each dataset is taken at slightly different times, for varying overall lengths of time, and especially for different sampling rates. To create a valid “averaged” estimate from a large number of datasets, each curve must be used independently to predict the response for a consistent set of input conditions. Implementing this approach first requires the user to find the range of “common” *Z*-Sensor values that all of the datasets contain, and generate a list of numbers between those values. This array will act as the “average *Z*-Sensor” in the following. Next, the *Z*-Sensor and the deflection vectors from each dataset are provided to a 1D interpolation function (such as “interp1()” in MATLAB), which accepts a sample set of *x*-values (the *Z*-Sensor dataset), a sample set of *y*-values (the deflection dataset), and a set of “query points” to evaluate the interpolation with. The average *Z*-Sensor array created previously will act as the “input” (i.e., query points) to interp1(), and the output will be the interpolated deflection values as predicted by a given dataset. This process continues for every AFM-SFS file used in the average, with each supplying a new set of *x*-values, a new set of *y*-values, and each being evaluated with the same input *Z*-Sensor array. A time array is then generated for each run by using the sampling frequency and number of points in that dataset as an estimate. Then, a spline is performed to re-sample the estimated time array such that it contains the exact same number of points as contained in the average *Z*-Sensor array. At the end of this step, the user can directly average the predicted deflection and time arrays from all datasets to obtain the “average deflection” and “average time”.

Clearly, when creating a list of numbers for the average *Z*-Sensor array at the beginning, it is critical to use an appropriate number of steps. Depending upon the number of points used, it is possible to artificially change the sampling rate to appear faster or slower, thus erroneously changing the temporal data contained within the averaged result. For example, if all of the individual experimental datasets contain roughly 1000 data points in the range of valid *Z*-Sensor values, and the average *Z*-Sensor array is made using an arbitrary 10,000 points, the average time vector will appear to have a timestep that is ten times faster than expected. This simple mistake can lead to the averaged dataset containing information that is not actually present in the force–distance files being processed. The number of points used to generate the average *Z*-Sensor array should be calibrated to match the number of points expected for a dataset of that length in the given experiment, such that the effective sampling frequency does not appear to change drastically. The most convenient means of verifying that the averaged dataset is valid involves looking at the average time array generated from the spline process, and ensuring that its timestep is close to the timestep of the individual data files. For example, if using a sampling frequency of 2 kHz for the AFM-SFS experiments, the average time array should appear to have a timestep of roughly 5 × 10^−4^ s.

#### Estimating material model parameters using a nonlinear least-squares approach

Modeling the force–indentation relationship with Equation 9 can be performed using a variety of techniques. In their paper, Lopez et al. utilize the cost-function minimization approach. The method involves comparing the left-hand side of Equation 9 to the predicted right-hand integral, which is calculated using a specific set of model parameters (*J_g_*, τ*_n_*, *J_n_*). These parameters are then varied by the built-in algorithm (specifically Levenberg–Marquardt in the case of Python’s minimize() function) to solve the nonlinear least-squares minimization problem. The function searches for the “optimal” parameter set that minimizes the relative error between the dataset and integral prediction.

Due to differences in the fit functions available, it is inconvenient to implement the Levenberg–Marquardt algorithm in MATLAB with the necessary upper and lower bounds on the model parameters. Instead, several nested “anonymous” functions (i.e., functions defined on a single line instead of in a separate file) are used to simulate a single experimental dataset. The top-level function is passed to lsqcurvefit(), which will call all of the sub-functions while fitting the parameters. The MATLAB function lsqcurvefit() is also capable of taking an input matrix as opposed to simply an array of data, meaning it could fit multiple datasets simultaneously if properly implemented. While this capability is not explored here, the ability to fit multiple averaged data sets from different experiments could prove useful in the future.

The series of nested anonymous functions are configured according to the following workflow:

1. *F*_conv_: Performs the convolution of 
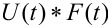
 using the “full” setting, resulting in an array that is *N* + *M* − 1 in length, where *N* is the number of points in *U*(*t*), and *M* is the number of points in *F*(*t*);
2. subref: Accepts the convolved result of *F*_conv_ and trims all data but a subset equal to the size of *t*_rep_;
3. *F*_conv,wrap_: Scales the output of subref by the time step d*t*, adds the glassy compliance term, and re-samples the result to logarithmic scaling. This is the function provided to lsqcurvefit().

Because lsqcurvefit() calculates the cost internally, it is not necessary to define a function for this purpose. However, other approaches may require an additional “comparison” function. The default algorithm used by lsqcurvefit() is “trust-region-reflective”, which is a nonlinear least-squares method, similar to Levenberg–Marquardt. A comparison of each approach and their efficacy is beyond the scope of this manuscript, but the reader is directed to the literature for explanations of both algorithms [27–28]. It is important to note that the parameter set obtained from these algorithms is not necessarily unique, and repeatedly performing the fit for a statistically significant number of times is recommended before evaluating its performance. Furthermore, the solutions are sensitive to the initial guess and parameter bounds. To counteract these effects the starting point for each parameter can be generated randomly within the upper and lower bounds for a fitting run, which is in turn performed many times to develop an understanding of the parameter space.

Beyond the function definitions and solver algorithms, it remains to appropriately determine and enforce the upper and lower bounds for each parameter, and also to choose starting “guesses” for each least-squares run. According to the specification of Lopez et al. [17], each successive term in the generalized-Voigt compliance function is intended to represent the material response during one order of magnitude in time. The time constants (τ*_n_*) are thus limited to a factor of ten lower and higher, with each additional term accounting for the next largest scale in the series. For example, a “one-term” series would contain the glassy compliance term and the compliance of a single Voigt element with a characteristic time centered of the order of the data acquisition frequency. Such a series would allow the characteristic time (τ_1_) to vary from 10^−5^ to 10^−3^ in the case of sampling at 10 kHz, and the compliance (*J*_1_) to be restricted to all reasonable values in the range (0,1). These terms should theoretically account for experiments with a maximum effective time of 0.01 to 1 ms, but for cases where the contact duration reaches 1 s or more this term alone would be insufficient to reproduce the material response. To adequately model these datasets, five or more terms would be necessary reaching characteristic times centered on 10^0^ or greater.

As mentioned previously, selecting an appropriate range of characteristic times is a critical step in extracting the most applicable parameter set. Without including a sufficient number of terms, the material response at large scales would dominate the lower orders, thus forcing each characteristic time parameter to tend toward their respective upper bounds. If such action is observed during the fit process, it is recommended to include an additional term and repeat the process until at most one order larger than the experiment length has been added, or the parameters are no longer converging on their upper or lower bounds.

It is evident from Equations 10–12 that each parameter set will also generate unique harmonic quantities. By examining the loss angle (δ(ω)), parameter groups could be evaluated and eliminated based upon their feasibility. In the case where a well-studied material is being investigated, this task can be relatively straightforward. However, for new materials without significant background literature it remains difficult to discard parameter sets based upon their harmonic quantities. The following section examines the fit procedure applied to nylon, and the effects of experimental settings on the quality of fit provided by the presented methodology.

### Extracting viscoelastic parameters from experimental data for nylon

A hollow nylon tube (source: McMaster-Carr, P/N 8628K48, Grade 6/6) was cut, ground smooth with a hand-held rotary tool, and cleaned in isopropyl alcohol (IPA) followed by a bath in deionized (DI) water. The sample was then allowed to dry sufficiently and mounted on an AFM metal specimen disk using double-sided carbon tape. The force mapping (six scan lines, six points per scan line) technique was implemented using an Asylum MFP-3D AFM to accommodate a variety of sample configurations, which yielded 36 indentation datasets at 36 different locations. Furthermore three approach velocities were selected in a logarithmic distribution: 10, 100, and 1000 nm/s. The probe utilized was an OLYMPUS AC 240TS-R3, featuring a tip radius of roughly 10 nm. Before measurement, the tip was calibrated using the thermal noise method [29] in which a hard silicon sample was used after sonicating using Mucasol, followed by IPA, and then DI water for a duration of 10 min per step. The resonant frequency and spring constant were found to be 68.953 kHz and 1.70 nN/nm, respectively.

**Figure 4:**
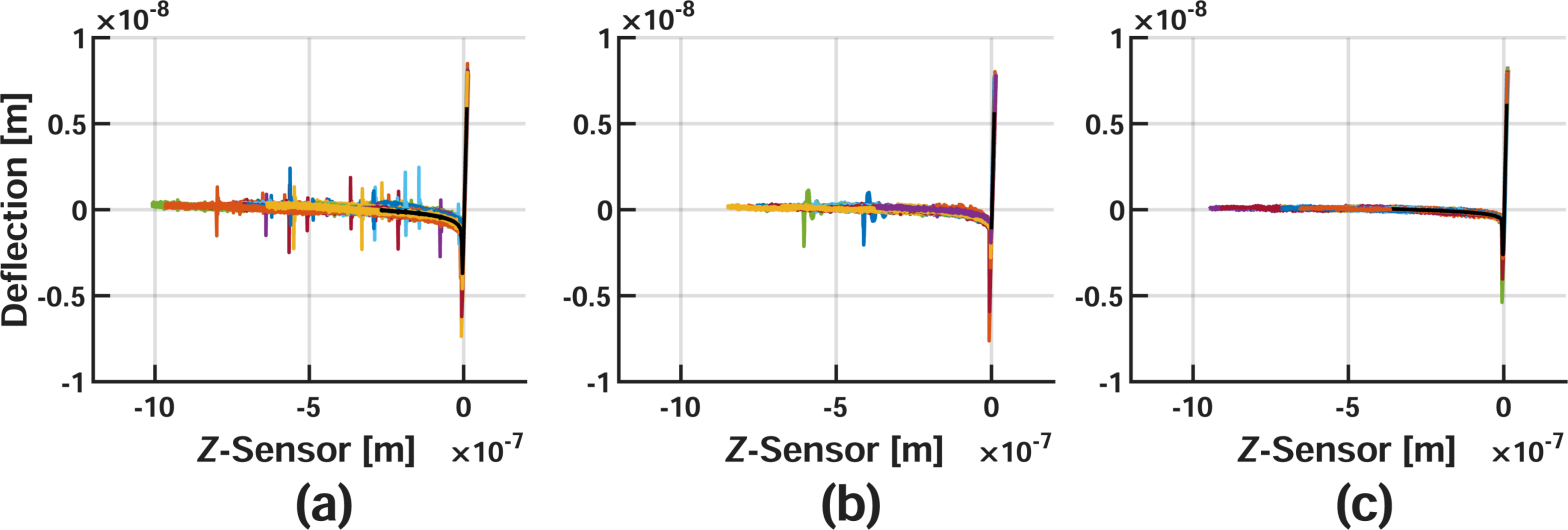
Conditioned AFM-SFS Data for different approach velocities. Corrected nylon data for (a) 10 nm/s, (b) 100 nm/s, and (c) 1000 nm/s. Colored lines represent the various datasets utilized for averaging, and the solid black lines plotted on top show the averaged result.

**Figure 5:**
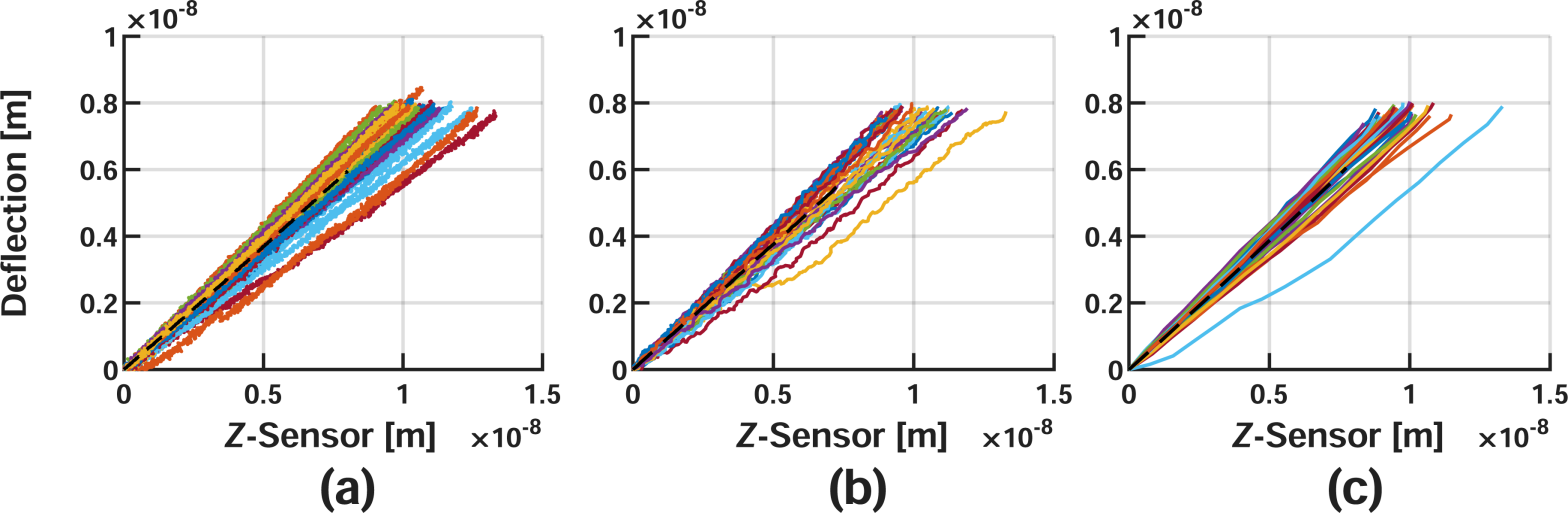
Repulsive (i.e., force application) portion of the curves in Figure 4 extracted for fitting. Corrected nylon data for (a) 10 nm/s, (b) 100 nm/s, and (c) 1000 nm/s. The colored lines represent various datasets included during the averaging process, and the dashed black lines show the averaged repulsive result. As the approach velocity decreases, the slope of each averaged dataset will also tend to decrease, meaning that less deflection occurs for a given *Z*-sensor reading. This is intuitive, as the material is given additional time to yield for slower approach velocities, thus appearing softer.

The curves are aligned as a result of the previously described conditioning steps for each velocity, and are visualized in Figure 4 and Figure 5. For clarity, the repulsive portions of each curve have also been isolated to show the exact data utilized for fitting Equation 9.

As expected, the fit qualities vary based upon the approach speed and number of fit terms used. This is due to varying amounts of long-timescale characteristics being present. For shorter experiments, using fewer terms resulted in a closer approximation while keeping characteristic time parameters away from their upper and lower bounds. The compliances generated in those cases are more reasonable, indicated by viscoelastic moduli of the order of several gigapascals, which agree with stiffnesses reported in the literature [30]. The parameter sets that were rated optimal for each approach velocity are given in Table 2.

**Table 2:** Generalized Kelvin–Voigt viscoelastic model parameter sets resulting from nonlinear least-squares fits of AFM-SFS force–indentation data. Characteristic times are given in units of [s], and compliances are provided in units of [Pa^−1^]. Note that the parameter sets utilized for generating the final fit are marked with an asterisk (*).

velocity [nm/s]	parameter	number of elements				

		1	2	3	4*	5

10	*J_g_*	3.76e-11	3.87e-12	3.79e-11	1.51e-11	2.65e-14

	*J*_1_	3.4e-09	2.22e-14	7.39e-11	3.5e-12	4.98e-12
	τ_1_	9.56e-04	2.22e-05	2.98e-04	5.1e-05	1.1e-04

	*J*_2_		3.94e-09	2.75e-10	1.09e-09	4.62e-10
	τ_2_		9.99e-03	1.01e-03	8.89e-03	4.53e-03

	*J*_3_			5.4e-09	2.11e-09	1.98e-09
	τ_3_			9.94e-02	7.55e-02	3.45e-02

	*J*_4_				8.24e-09	7.51e-09
	τ_4_				7.52e-01	5.01e-01

	*J*_5_					2.49e-09
	τ_5_					8.05

		1	2	3*	4	5

100	*J_g_*	4.86e-14	2.88e-13	2.15e-11	6.14e-11	1.87e-10

	*J*_1_	2.8e-09	2.22e-14	1.06e-10	3.2e-14	3.16e-10
	τ_1_	9.71e-04	2.32e-04	7.58e-04	5.15e-04	9.89e-04

	*J*_2_		4.63e-09	3.27e-10	5.1e-10	7.05e-11
	τ_2_		9.99e-03	6.33e-04	1.06e-03	8.13e-03

	*J*_3_			4.94e-09	4.61e-09	4.68e-09
	τ_3_			1.72e-02	1.7e-02	1.66e-02

	*J*_4_				4.66e-09	2.23e-09
	τ_4_				7.45e-01	9.02e-01

	*J*_5_					3.02e-09
	τ_5_					3.76

		1	2	3*	4	

1000	*J_g_*	5.1e-14	3.73e-14	3.65e-11	2.48e-14	

	*J*_1_	3.18e-09	1.24e-09	1.64e-09	6.92e-10	
	τ_1_	9.96e-04	5.82e-04	7.35e-04	9.7e-04	

	*J*_2_		7.64e-09	2.22e-14	9.77e-10	
	τ_2_		9.46e-03	6.26e-03	6.11e-04	

	*J*_3_			3.51e-08	1.71e-08	
	τ_3_			6.08e-02	3.85e-02	

	*J*_4_				1.25e-07	
	τ_4_				9.3e-01	

**Figure 6:**
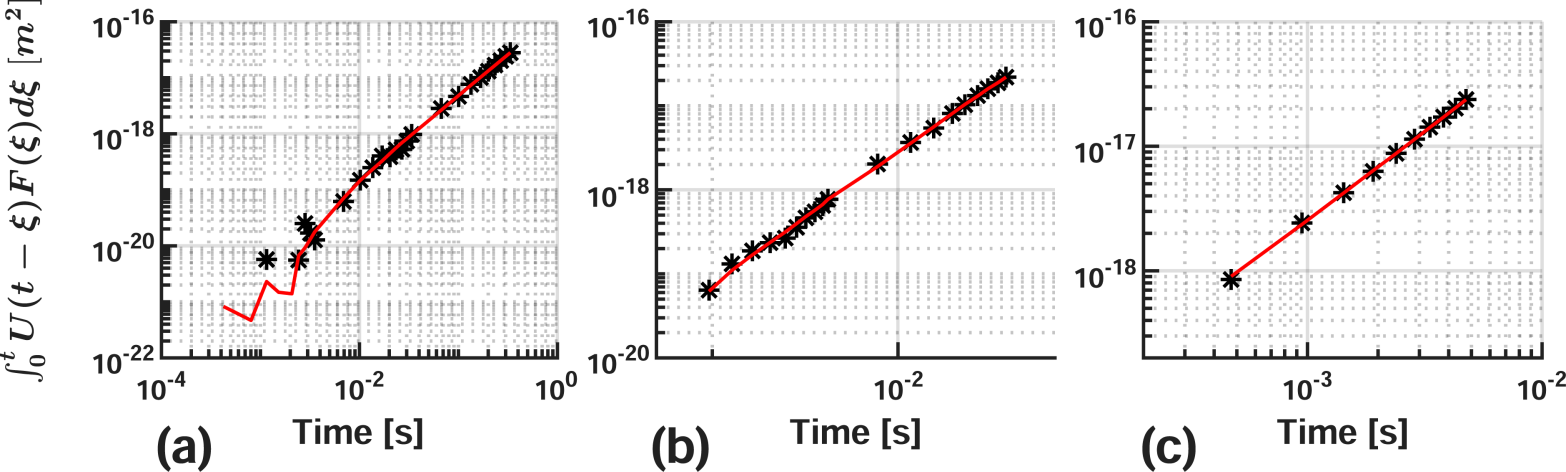
AFM-SFS data and parameterized generalized-Voigt mechanical model. Data and nonlinear least-squares fit for (a) 10 nm/s, (b) 100 nm/s, and (c) 1000 nm/s. The parameters utilized in each case have been marked in Table 2 with an asterisk (*). The results are comparable to those noted by Lopez and co-workers [17].

After evaluating the fit quality for each velocity, it was determined that four terms provided the best data approximation for the 10 nm/s dataset, while three terms were sufficient for 100 and 1000 nm/s. Figure 6 shows the fitted viscoelastic model against each corresponding dataset. Note that the parameterization was unsuccessful for the 5-Voigt series fit on the 1000 nm/s averaged data because the conditioned repulsive dataset contained less than eleven data points. This is significant because lsqcurvefit() will not operate on models where the number of parameters within (eleven terms for the 5-Voigt series) is greater than the number of data points used for comparison.

**Figure 7:**
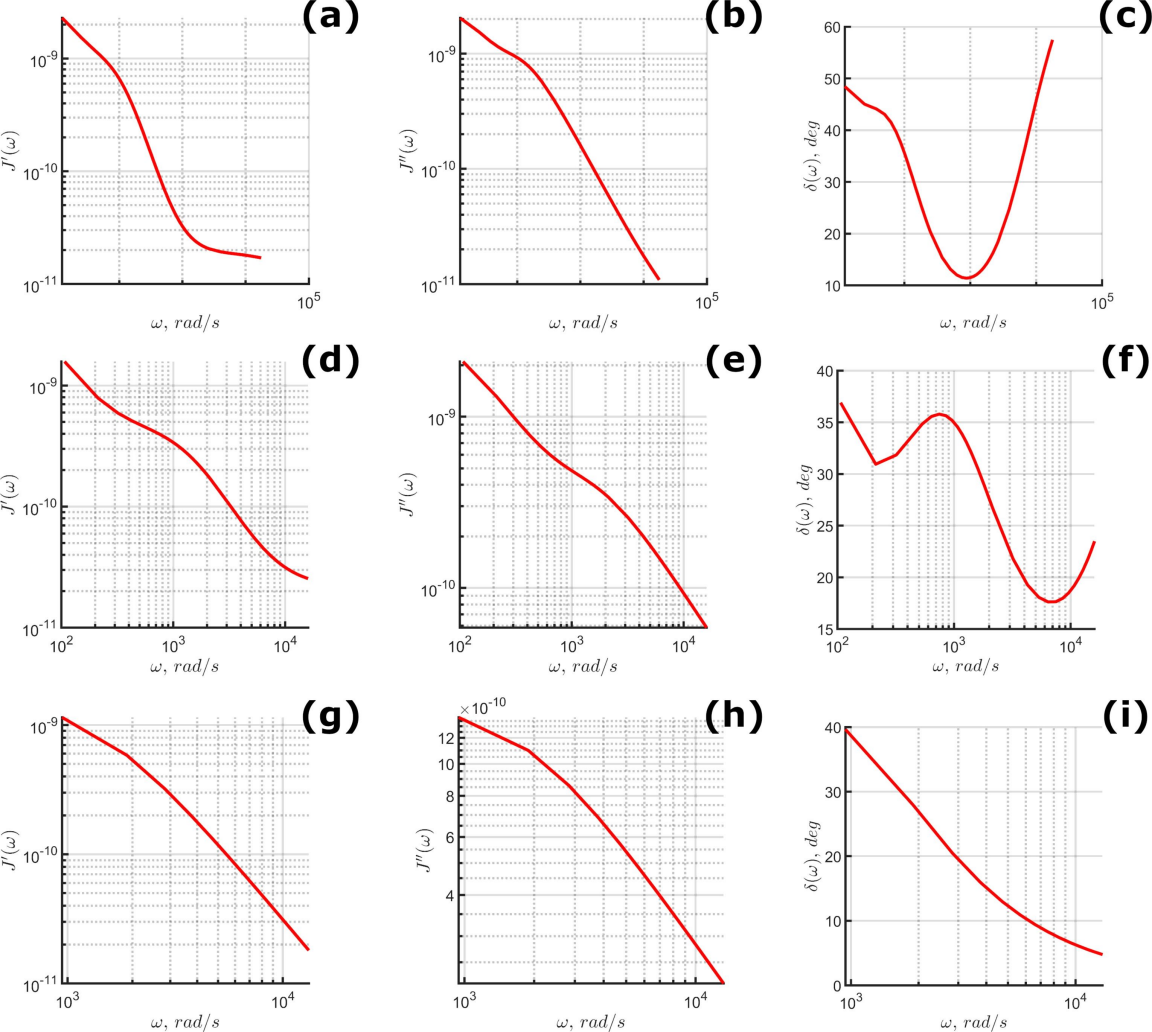
Predicted harmonic quantities for 10 nm/s (top row), 100 nm/s (middle row), and 1000 nm/s (bottom row) approach velocity. (a, d, g) Calculated storage compliance; (b, e, h) calculated loss compliance; (c, f, i) calculated loss angle.

**Figure 8:**
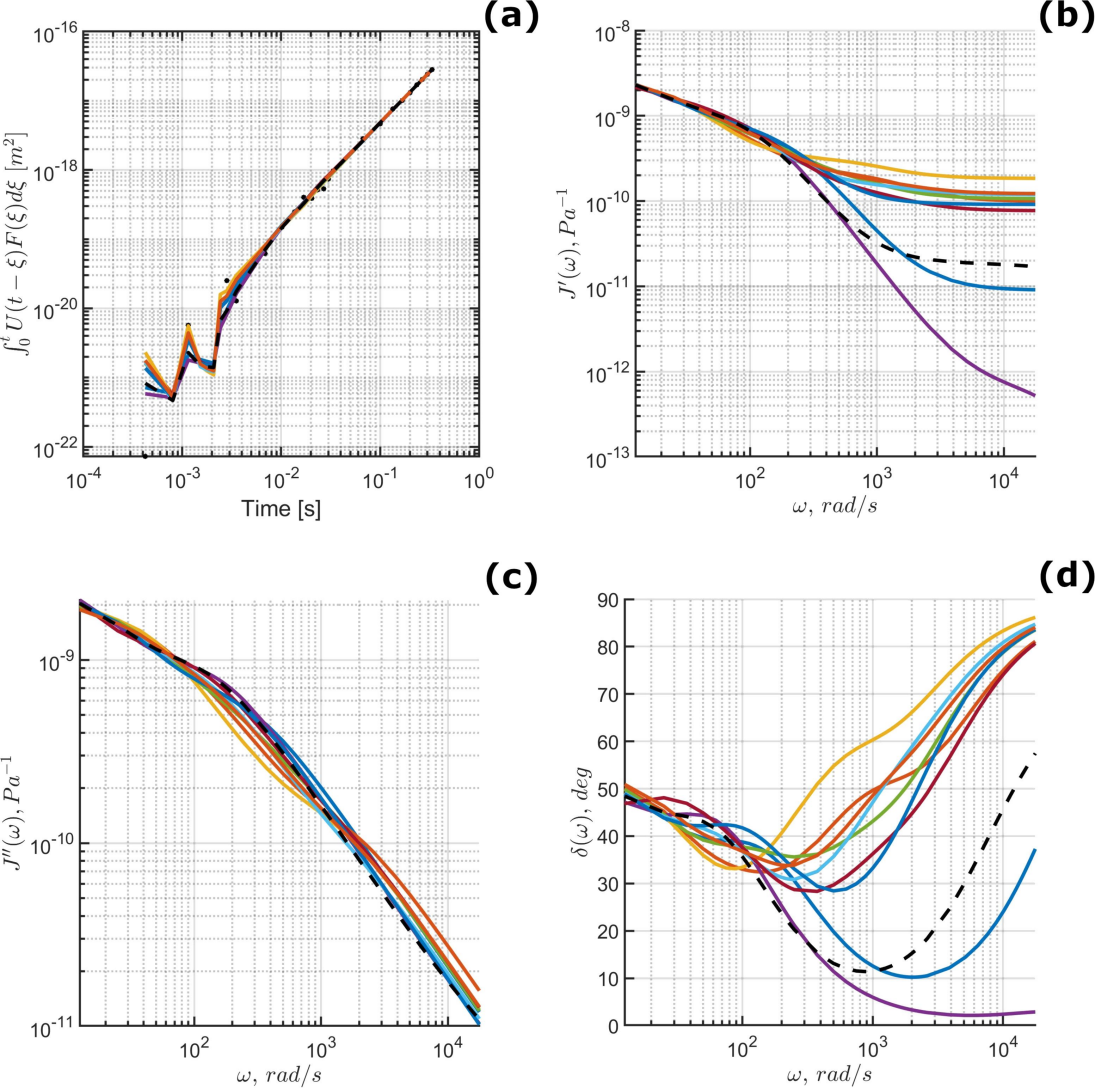
(a) Data fit, (b) storage compliance, (c) loss compliance, and (d) loss angle, calculated for the optimized material models parameter sets ranked 1–10 based upon normalized residual; 10 nm/s approach velocity. Note that the highest ranked fit is given by a dashed black line and the colored lines represent the ranks 2–10. Clearly there can be significant differences in the predicted harmonic quantities while providing a close approximation of the experimental data in panel (a). The loss angle variances shown in panel (d) are driven by significant changes in the storage compliance, shown in panel (b).

Harmonic quantities have also been calculated using the fit parameters from Figure 6, providing the predicted relationship between the elastic and viscous material response. The results are provided in Figure 7. It is critical to reiterate that each set of optimization parameters is not unique. There is a theoretically unlimited number of distinct parameter groupings that could recreate the material response for a given experiment. With each parameter set, the calculation of Equations 10–12 will indicate different distributions for the observed harmonic quantities. To illustrate the possible variations, Figure 8 visualizes the predictions generated by the top-ten ranked parameter sets and their apparent fit to the averaged 10 nm/s approach velocity dataset.

Clearly, estimating the harmonic properties of a material sample can be difficult using a single AFM-SFS experiment. As the number of test sites on the sample surface increases, the larger data population should theoretically limit the number of suitable parameter sets that recreate all of the material responses. Similarly, it could also be worthwhile to attempt to fit an even larger number of datasets using a variety of approach velocities simultaneously. Considering multiple approach velocities would reduce the parameter space, eliminate the need for a comparison of the results between approach velocities, and could provide a more generally applicable set of parameters after one script iteration. The implementation of a multiple-velocity fit process would be significantly more complex, and would require dynamically eliminating model terms for different velocities, such that each dataset would be compared to a corresponding model with an appropriate number of characteristic times. To avoid adding this additional degree of freedom to the problem, it would be necessary to have a priori knowledge of the optimal number of terms to use for each velocity. This would remove the need to vary the number of terms dynamically and drastically reduce the associated computational overhead. Regardless of the approach, determining an effective method for extracting the most broadly applicable parameters, and by extension their corresponding harmonic quantities, remains an attractive subject for further research.

## Conclusion

A methodology for extracting viscoelastic parameters from a sample material response in AFM-SFS experiments has been discussed and applied to the specific case of nylon 6,6 using multiple approach velocities. Beginning with the correspondence principle for the spherical indentation of a viscoelastic half-space, the presented inversion approach has been shown to provide mechanical and temporal insight into the viscoelastic response of nanoscale surfaces. As stated previously, the assumptions utilized during the objective function derivation places inherent limits on the appropriate experimental settings. Most critically, the indentation strain measures must remain small to prevent violation of the underlying theory. Common pitfalls and limitations have been discussed, with particular emphasis on both data conditioning and mechanical-equivalent model design. One important issue discussed was that the number of characteristic terms included in compliance series models must be intentionally prescribed based upon the length and data acquisition rate of a given experiment. If the effects of experiment length and the desired application time range are not considered, an otherwise appropriate model could be incorrectly parameterized and perform poorly, or be applied outside its valid temporal range. There remain many attractive questions worthy of investigation, including further restriction of the best-fit parameters using their implied harmonic quantities, extending the theoretical implementation to include multi-axial datasets, and the application of this inversion approach to nonlinear compliance models – the last of which is the subject of a future paper by the authors.

## Supporting Information

The Supporting Information includes a directory containing all necessary MATLAB scripts used for AFM-SFS file inversion, and an instructional document to explain the operation and settings available in the script. Additionally, the full derivations mentioned in section “Theoretical Background” are included in Supporting Information File 1.

File 1 Readme file for MATLAB script operation.

File 2 Compressed directory containing the MATLAB script and supporting functions.

File 3 More detailed derivations of the relationships presented in section ‘‘Theoretical Background’’.
