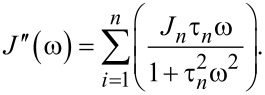# Correction: Extracting viscoelastic material parameters using an atomic force microscope and static force spectroscopy

**DOI:** 10.3762/bjnano.12.10

**Published:** 2021-01-28

**Authors:** Cameron H Parvini, M A S R Saadi, Santiago D Solares

**Affiliations:** 1 Department of Mechanical and Aerospace Engineering, The George Washington University School of Engineering and Applied Science, 800 22nd St. NW, Suite 3000, Washington, DC 20052, United States

**Keywords:** atomic force microscopy (AFM), creep, force mapping, indentation, Kelvin–Voigt, static force spectroscopy (SFS), viscoelasticity

In the “Useful Viscoelastic Quantities” section of the original publication, it is stated that the storage modulus (*E*′) and storage compliance (*J*′) are inverses of one another (Equation 10). Similarly, it is stated that the loss modulus (*E*″) and loss compliance (*J*″) are inverses of one another (Equation 11). However, it is the relaxance (*Q*) and retardance (*U*) that are inverses of one another *in the Laplace domain* (not in the time domain), leading to a more complex relationship between the moduli and their respective compliances. Translation between harmonic quantities can be accomplished through the expressions below, where 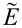 is the absolute modulus and 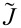 is the absolute compliance [1]:

[1]
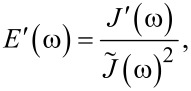


[2]
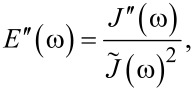


[3]
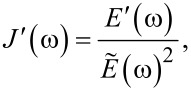


[4]
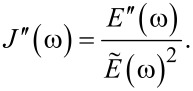


Absolute modulus and absolute compliance are calculated as:

[5]
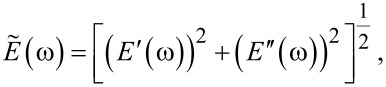


[6]
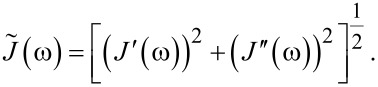


The leftmost term in Equation 10 and Equation 11 in the original manuscript is thus incorrect and needs to be removed, leaving the following corrected expressions:

Equation 10:


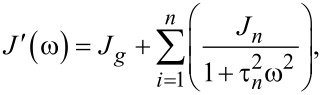


Equation 11: