# Supplementary material for: Extracting viscoelastic material parameters using an atomic force microscope and static force spectroscopy
Source: Beilstein J Nanotechnol. 2020 Jun 16;11:922–37. doi: 10.3762/bjnano.11.77 (PMC7308608; doi:10.3762/bjnano.11.77)
Supplement: File 1 — Readme file for MATLAB script operation. [file Beilstein_J_Nanotechnol-11-922-s001.pdf]

BEILSTEIN JOURNAL OF NANOTECHNOLOGY

Supporting Information

for

Extracting viscoelastic material parameters using an atomic force microscope and static force spectroscopy

Cameron H. Parvini, M. A. S. R. Saadi and Santiago D. Solares

Beilstein J. Nanotechnol. **2020,** *11,* 922–937. doi:10.3762/bjnano.11.77

Readme file for MATLAB script operation

License and Terms: This is a supporting information file under the terms of the Creative Commons Attribution License (http://creativecommons.org/licenses/by/4.0). Please note that the reuse, redistribution and reproduction in particular requires that the authors and source are credited.
The license is subject to the *Beilstein Journal of Nanotechnology* terms and conditions: (https://www.beilstein-journals.org/bjnano)

AFM SFS Viscoelastic Parameter Inversion Script (MATLAB)

The scripts included in this directory will perform the data conditioning and analysis required to fit viscoelastic model parameters to Atomic Force Microscope (AFM) Static Force Spectroscopy (SFS) datasets. They have been submitted as supplemental materials for a manuscript, titled "Extracting Viscoelastic Material Parameters using an Atomic Force Microscope and Static Force Spectroscopy". For further details on the methods and analytical justifications, please direct your attention to that paper.

Prerequisites

- MATLAB (>= 9.4)
- MATLAB Signal Processing Toolbox
- MATLAB Parallel Computing Toolbox
- MATLAB Curve Fitting Toolbox
- MATLAB Optimization Toolbox

Running the Script

First, the primary MATLAB script (ParameterExtraction.m) must be opened and run.

Second, the user will be prompted to select the directory where their AFM SFS files reside. These files must either follow one of the pre-programmed formats, or the user must implement an additional "case" for the switch functions on lines 45 and 83. The filenames must contain the approach velocity, position number, and run number for that particular dataset. For example, when using one of the built-in naming conventions, the filenames could be formatted as such:

- FD_**Point Number_Run Number_Velocity**.ibw
- ELopez_**Point Number_Run Number_Velocity**.ibw
- ELopezSim_**Point Number_Run Number_Velocity**.txt

The underscore-separation and variable location must be kept consistent. In addition, the user should replace periods "." in the velocity with hyphens "-" to separate integers from decimals. If a different filetype needs to be used, the user must evaluate which functions are available in MATLAB natively or on the MATLAB exchange to open that filetype and extract the raw data.

The purpose for including ELopez and ELopezSim is for the user to utilize supplemental data and simulation files provided by Lopez et al. with their manuscript, which can be found on Github here.

A test directory has been included here (/TestFiles/), which contains properly formatted filenames for experiments with an approach velocity of 1000 nm/s.

Settings

Currently, the script generates figures but does not save them programmatically. There are a variety of points where the authors have included "saveas" and "savefig" functions to allow users to save their matlab figures in a local directory, called TempFigs. If the user does not wish to save in TempFigs, an alternative link must be provided to these functions and the directory must already exist before running the script.

Once the data has been initally conditioned and then plotted for clarity, the user will be prompted to enter a series of values:

"Please enter the radius of the indenter in nanometers"

This value must be an integer, and be in the range 0 < r < 10,000.

If the input is not valid (for this and other settings), the user will be prompted to enter a new, valid setting. For a 10 nm tip radius, the user would simply type "10" and hit enter to continue.

"Please enter the number of voigt terms you would like to use (1-5)"

This value must be an integer, and be in the range 1 <= n <= 5.

This script has been geared towards utilizing a generalized voigt series for fitting. While it is certainly possible to implement the cases for additional terms, it would require additional customization of the models and knowledge of how to program nested anonymous functions for use with lsqcurvefit().

"Would you like to run this file for each Voigt term number below your selection? (i.e. for 5-term, this will run for 1, 2, 3, and 4 terms, too) (y/n)"

This value must be a character, and be either "y" or "n".

If active ("y"), the simulation will run for each number of voigt terms up to and including the input given for the previous setting. Essentially, if the user specified 5 voigt terms previously and activated this setting, the simulation would run for 1 term, 2 terms, and so on until 5 terms had been simulated. The data for each fitting would be saved in a separate matlab file (.mat format) for analysis in MATLAB later.

"Would you like to skip any numbers? If so, please enter them in array form here, or use empty brackets ([])"

This value must be an array of integers, i.e. a list of numbers separated by spaces ( ) or commas (,) surrounded by square brackets. For example, to skip simulations for 1 and 2 terms, the user would enter: [1 2]

This setting allows users to skip simulation loops for specific term numbers. This is only relevant if "y" was provided to the previous setting. In the case of the 5 voigt series setting, to be run for each number below, this setting skips simulations for 1 and 2 voigt terms.

"Are you using individual (i) or averaged analysis(a)?"

This value must be a character, and be either "i" or "a".

This setting is critical: if set to individual analysis, the script will run the voigt model fit for every single file individually. This is useful if the user is unsure whether there are significant outliers in the dataset. If set to use averaged analysis, the script will run once for the "average dataset" described in the manuscript, which is a dataset created using every file in the directory of that approach velocity. Averaged analysis is recommended.

"What scaling factor would you like to use on the fitting tolerances?"

This value must be an integer, and be in the range 0 < x < 1.

This value is somewhat difficult to prescribe. It equates to the tolerance provided to lsqcurvefit during the fitting process. For the accompanying manuscript, an "accuracy" or "tolerance" of 1e-40 was utilized for all approach velocities. This value should be decreased if the fit quality is poor, likely caused by significantly smaller forces, or if the fit is poor and forces are large.

"Would you like to save .mat files? (y/n)"

This value must be a character, and be either "y" or "n".

Saves intermediary .mat files throughout the course of simulation. Useful only if there are concerns about the script not working properly (i.e. primarily for debugging). The script will always save a single .mat file at the end of a simulation run for a given velocity or individual dataset.

Manuscript Authors

- **Cameron Parvini** - *cparvini@gwmail.gwu.edu* [Primary Contact for Coding Questions] - LinkedIn
- **M.A.S.R. Saadi** - *abid_saadi@gwmail.gwu.edu*
- **Santiago Solares** - *ssolares@gwmail.gwu.edu* [Corresponding Author] - Google Scholar

See also the website for our Scanning Probe Microscopy Lab, which includes current and former contributors.

Acknowledgments

A sincere thank you to Dr. Enrique Lopez for all of his assistance in designing and troubleshooting the code supplied here.
