# Supplementary material for: Extracting viscoelastic material parameters using an atomic force microscope and static force spectroscopy
Source: Beilstein J Nanotechnol. 2020 Jun 16;11:922–37. doi: 10.3762/bjnano.11.77 (PMC7308608; doi:10.3762/bjnano.11.77)
Supplement: File 3 — More detailed derivations of the relationships presented in section ‘‘Theoretical Background’’. [file Beilstein_J_Nanotechnol-11-922-s003.pdf]

BEILSTEIN JOURNAL OF NANOTECHNOLOGY

Supporting Information

for

Extracting viscoelastic material parameters using an atomic force microscope and static force spectroscopy

Cameron H. Parvini, M. A. S. R. Saadi and Santiago D. Solares

Beilstein J. Nanotechnol. **2020,** *11,* 922–937. doi:10.3762/bjnano.11.77

More detailed derivations of the relationships presented in section ‘‘Theoretical Background’’

License and Terms: This is a supporting information file under the terms of the Creative Commons Attribution License (http://creativecommons.org/licenses/by/4.0). Please note that the reuse, redistribution and reproduction in particular requires that the authors and source are credited.
The license is subject to the *Beilstein Journal of Nanotechnology* terms and conditions: (https://www.beilstein-journals.org/bjnano)

Spherical Indentation of a Viscoelastic Half-Space

In an ideal case, the exact tip geometry is known a priori—in lieu of such information, it is expedient to make simplifying assumptions about the tip shape at the point of contact. One common assumption is that the local surface deformations are small, such that the tip has a roughly spherical contact geometry during indentation. This assumption is best made for larger diameter tips or specialized colloidal probes, although it can be argued that smaller tips can still be approximated provided the indentation depth is small. Lee and Radok [1] proposed a solution to the rigid spherical contact of a viscoelastic half-space nearly sixty years ago. The indentation configuration is visualized in Figure S1, which is based upon Figure 1 in their original manuscript.

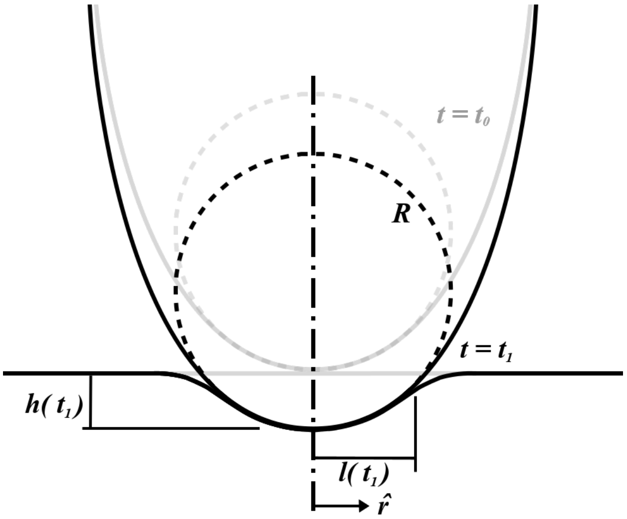

Figure S1: The quasi-static spherical indentation configuration as outlined by Lee and Radok in [1].

Here, the deepest indentation occurs at the center of the sphere ($r = 0$) and is labeled $h(t)$. The indenter has a radius of curvature R, and the distance from the center axis to the edge of contact, known as the contact radius, is labeled $l(t)$.

Two terms must also be introduced to describe the physical response of viscoelastic materials. First, "retardation" is the delayed deformation of a material under an applied force or stress, as seen most commonly in polymers. Second, "relaxation" refers to the gradual decrease in stress under a constant applied deformation [2]. The material retardance ($\bar{U}$) and relaxance ($\bar{Q}$) are similarly a series of linear operators in time that can take the form of differentials, algebraic descriptions of spring-dashpot

mechanical models, or hereditary integral operators, all of which can approximate the viscoelastic retardation or relaxation of the material. When defining the Laplace domain stress ($\bar{\sigma}(s)$) or strain ($\bar{\epsilon}(s)$), the relaxance or retardance can be used to convert between the stress and strain. The form of $\bar{U}$ and $\bar{Q}$, and by extension their constituent linear operator terms $\bar{u}$ and $\bar{q}$, is a point of discussion later. For now they will be represented in equations as time-dependent operators of which the relationships in the Laplace domain are:

$$\bar{\sigma}(s) = \bar{Q}\bar{\epsilon}(s), \qquad \bar{Q} = \frac{\bar{q}}{\bar{u}} \qquad \text{“Relaxance”} \tag{S1}$$

$$\bar{\epsilon}(s) = \bar{U}\bar{\sigma}(s), \qquad \bar{U} = \frac{\bar{u}}{\bar{q}} \qquad \text{“Retardance”} \tag{S2}$$

In their solution, Lee and Radok specify the following boundary conditions:

1. The sphere is smooth, thus excluding adhesion and tangential forces;

2. the sphere is also rigid, meaning it will not deform throughout the contact;

3. the half-space is initially planar, and undisturbed;

4. the viscoelastic stress-strain relations take the linear, isotropic form. The stress tensor is split between principal (σ_{ii}) and deviatoric (s_{ij}) components, and similarly with the principal strain (ϵ_{ii}) and deviatoric strain (e_{ij}). In an analogous fashion, the time-domain linear viscoelastic operator terms ($u(t)$,$q(t)$) are split between principal (u',q') and deviatoric (u,q) components. The stress–strain relationships thus take the form:

$$us_{ij} = qe_{ij} \tag{S3}$$

$$u'\sigma_{ii} = q'\epsilon_{ii} \tag{S4}$$

5. The boundary condition outside of the contact area ($r > l(t)$) is zero surface traction;

6. The boundary condition within the contact area ($r \leq l(t)$) is a normal displacement defined by the geometry of the indenter.

By utilizing the Hertzian contact solution, an additional pair of requirements are implicit in Lee and Radok's derivation:

1. The strain is small, allowing the continuum mechanics strain tensors to be simplified;

2. the solution is Quasi-static, meaning that time only appears in the equations such that the current value of the contact radius $l(t)$ can be referenced.

The first Hertzian restriction is commonly applied in continuum mechanics derivations to enforce the linearization of strain [3]. This condition is used for mathematical convenience, and affects both translation between the Eulerian (deformed) and Lagrangian (initial) strains, and the approach to simplifying the Clausius–Duhem inequality. Strain linearization causes the second-order (i.e. nonlinear) terms to be removed from the strain tensors, meaning that the difference between the Eulerian and Lagrangian strains is isolated to differences in the coordinate systems. For small strains and rotations, these differences are subsequently negligible and the two reference frames coincide. Similarly, neglecting the nonlinear strain simplifies the rate of deformation tensor (D) to the extent that it can be replaced by the Green–Lagrange strain rate tensor ($\dot{E}$) in the Clausius–Duhem inequality. This step removes the need to calculate the spatial velocity gradients, which can be difficult to measure, at the expense of limiting the range of applicable strains and rotations for the resulting continuum relationships. One must determine the suitability of strain linearization to a given problem before utilizing the approach described by Lee and Radok. The small strain assumption most commonly holds for cases where both the displacement vector and displacement gradient are significantly smaller than unity. By extension, within the context of AFM, this will restrict the indentation strain to small numbers, and place inherent limitations upon any derived relationships. Also note that the second Hertzian requirement means the boundary conditions of the problem will change in time, since at each new time instance a point that was previously outside of contact area moves within the contact radius $l(t)$, and therefore changes its boundary condition. The

Hertzian solution for spherical contact of an elastic half-space takes the form:

$$p(r,t) = \frac{4}{\pi R}\Big(\frac{G}{1-\nu}\Big)Re\big[l(t)^2 - r^2\big]^{1/2} \tag{S5}$$

where the shear modulus G and Poisson's radio ν are sufficient to prescribe the pressure distribution $p(r,t)$ in space and time. From Equation S5, Lee and Radok argue that because the shear modulus is a material parameter that converts shear strain into shear stress, it can be replaced by a corresponding viscoelastic counterpart. In terms of the shear stress s_{ij} and shear strain ϵ_{ij}:

$$\begin{aligned} us_{ij} &= qe_{ij} \\ s_{ij} &= \frac{q}{u}e_{ij} \\ G &\approx \frac{q}{u} \end{aligned} \tag{S6}$$

For the case of an incompressible material, Poisson's ratio is also taken to be $\frac{1}{2}$, further simplifying Equation S5 and replacing the shear modulus term. This condition is not specifically required, but is taken for simplicity during the Lee and Radok derivation [1]. The viscoelastic equivalent of Equation S5 is therefore:

$$p(r,t) = \frac{8}{\pi R}\big(\frac{q}{u}\big)Re\big[l(t)^2 - r^2\big]^{1/2} \tag{S7}$$

The authors further reduce Equation S7 by replacing the radical term with a new function definition $f(r,t)$. This occurs purely for mathematical simplicity, since the function $f(r,t)$ will eventually be transformed several times to and from Laplace space. The notation would become more complex if the function was left in its real-operator radical form. It is most important to remember that $f(r,t)$ will only be nonzero inside of the contact area (i.e., $r < l(t)$). In its fully reduced form, the pressure distribution of a viscoelastic half-space is then transformed to Laplace space.

$$\mathcal{L}\Big[u[p(r,t)] = \frac{8}{\pi R}q[f(r,t)]\Big] \tag{S8}$$

$$\bar{p}(r,s) = \frac{8}{\pi R}\frac{\bar{q}(s)}{\bar{u}(s)}\bar{f}(r,s) \tag{S9}$$

Here, the quantity $\bar{p}(r,s)$ represents the Laplace transform of the time domain pressure distribution $p(r,t)$. To continue with the derivation, it becomes necessary to describe the surface displacement. Integrating the surface pressure distribution presented in Equation S9 using the point-load solution from elastic theory, Lee and Radok further limit the contact region to some "reasonable" limit A_{max}. This allows enforcing static spatial boundary conditions for the surface pressure integration, and due to the real operator on the radical in $f(r,t)$, the function value will remain zero outside of the contact radius $l(t)$ at any given time. By specifying l_{max} to be some arbitrary number sufficiently larger than the expected maximum contact radius, the entire solution will be contained within the bounds of integration. Writing the Laplace form of the displacement distribution:

$$\bar{w}(r,t) = \iint_{A_{max}} \frac{1}{2\pi}\frac{\bar{u}(s)}{\bar{q}(s)}\frac{\bar{p}(r',s)}{\rho}dA \tag{S10}$$

Here, the variable ρ is the distance from a normal axis passing through the center of the spherical indenter to a differential area element dA, with r' being the radius coordinate being integrated across the contact surface. Substituting Equation S9 into Equation S10 for $\bar{p}(r,s)$ gives:

$$\begin{aligned}
\bar{w}(r,t) &= \iint_{A_{max}} \frac{1}{2\pi}\frac{\bar{u}(s)}{\bar{q}(s)}\frac{\bar{p}(r',s)}{\rho}dA \\
\bar{w}(r,t) &= \iint_{A_{max}} \frac{1}{2\pi}\frac{\bar{u}(s)}{\bar{q}(s)}\frac{8}{\pi R}\frac{\bar{q}(s)}{\bar{u}(s)}\bar{f}(r',t)\frac{1}{\rho}dA \\
\bar{w}(r,t) &= \iint_{A_{max}} \frac{4}{\pi^2 R}\frac{\bar{f}(r',s)}{\rho}dA
\end{aligned} \tag{S11}$$

Due to the fact that fixed integration limits were enforced by specifying A_{max}, the inverse Laplace operator can be moved inside of the integral without issue. Importantly, the definition for $f(r,t)$ conveniently allows the bounds of integration to be re-specified since $f(r,t) = 0$ for $r' \geq l(t)$. Thus,

the displacement for $r \leq l(t)$ can be found as follows:

$$\mathcal{L}^{-1}\left[\bar{w}(r,t) = \iint\limits_{A_{max}} \frac{4}{\pi^2 R}\frac{\bar{f}(r',s)}{\rho}dA\right]$$
$$w(r,t) = \frac{4}{\pi^2 R}\iint\limits_{A_{max}} \frac{f(r',t)}{\rho}dA$$
$$= \frac{2[l(t)]^2}{R} - \frac{r^2}{R} \tag{S12}$$

Equation S12 can be utilized to re-cast the description of $f(r,t)$ in terms of a more useful quantity: the central displacement of the indenting sphere. By evaluating Equation S12 at $r = 0$:

$$h(t) = \frac{2[l(t)]^2}{R} \tag{S13}$$

Similarly, the pressure distribution can be manipulated to depend upon the total penetration force—a more easily measured experimental quantity. In an analogous approach to enforcing constant boundary conditions above, the pressure distribution is integrated to acquire the total force $F(t)$ and manipulated in the Laplace domain to allow mobility of the operators u and q. The process is as follows:

$$F(t) = \int_0^{l(t)} p(r,t)2\pi r dr \tag{S14}$$
$$\mathcal{L}\left[F(t) = \int_0^{l(t)} p(r,t)2\pi r dr\right]$$
$$\bar{f}(s) = \int_0^{l_{max}} \mathcal{L}[p(r,t)]2\pi r dr$$
$$= \int_0^{l_{max}} \mathcal{L}[\frac{8}{\pi R}\frac{q}{u}f(r,t)]2\pi r dr$$
$$= \frac{8}{R}\frac{\bar{q}(s)}{\bar{u}(s)}\int_0^{l_{max}} \bar{f}(r,s)2r dr$$

$$\mathcal{L}^{-1}\left[\bar{f}(s) = \frac{16}{R}\frac{\bar{q}(s)}{\bar{u}(s)}\int_{0}^{l_{max}} \bar{f}(r,s)rdr\right]$$

$$u[F(t)] = \frac{16}{R}q[\int_{0}^{l(t)} f(r,t)rdr]$$

$$= \frac{16}{R}q[\int_{0}^{l(t)} (\sqrt{Rh(t) - r^2})rdr]$$

Substituting $x = \frac{1}{2}Rh(t) - r^2$, and $dx = -2rdr$:

$$= \frac{16}{R}q[\int_{0}^{l(t)} -\frac{1}{2}\sqrt{x}dx]$$

$$= \frac{-16}{2R}q[\frac{x^{3/2}}{3/2}\Big|_{0}^{l(t)}]$$

$$= \frac{-16}{3R}q[(\frac{1}{2}Rh(t) - r^2)^{3/2}\Big|_{0}^{l(t)}]$$

$$= \frac{-16}{3R}q[-l(t)^3]$$

$$u[F(t)] = \frac{16}{3R}q[l(t)^3] \qquad \text{OR:} \tag{S15}$$

$$u[F(t)] = \frac{16\sqrt{R}}{3}q[\{h(t)\}^{3/2}] \tag{S16}$$

Equation S16 represents the relationship between applied load and spherical indentation depth for a viscoelastic material having characteristic operators u and q. It is the relationship upon which Lopez et al. build a solution for the AFM experiment, and is originally presented as Equation 3 in their paper [4].

Extending the Solution to Arbitrary Load History

Traditionally, when using creep-recovery experiments to parameterize the viscoelastic models under study, a constant stress is first applied to a sample then removed later on. For example, one method

involves hanging weights from a material for some time, then removing the weights and allowing the material to recover. A strain gage or displacement sensor captures the deformation occurring during both phases, and the data is used for fitting. The boundary conditions for such an approach dictate a step-function in stress, and that one end of the sample is fixed. In that case, the constant load history would suggest using the "creep compliance" $J(t)$, an engineering quantity that represents the change in strain as a function of time for a medium subjected to an instantaneous constant stress [1,4]. While applicable for this style of study, the tip–sample interaction during AFM experiments does not apply a constant force (i.e., stress) in time—the load history is more reminiscent of a discrete impulse function, where the contact time is short. As such, while the solution form could use the creep compliance for fitting, it is more direct to use the material retardance. Before discussing the benefits, the creep compliance is defined in terms of the applied stress $\sigma(t)$, strain $\epsilon(t)$, and material retardance $\bar{U}(s)$:

$$J(t) = \frac{\epsilon(t)}{\sigma_0} \qquad \text{for} \qquad \sigma(t) = \sigma_0 \tag{S17}$$

$$\bar{J}(s) = \frac{\bar{U}(s)}{s} \tag{S18}$$

By taking the Laplace transform of Equation S16 and rearranging:

$$\begin{aligned}
\mathcal{L}[\frac{3}{16\sqrt{R}}u[F(t)] &= q[\{h(t)\}^{3/2}]] \\
\frac{3}{16\sqrt{R}}\bar{u}(s)\bar{f}(s) &= \bar{q}(s)\mathcal{L}\left[\{h(t)\}^{3/2}\right] \\
\frac{3}{16\sqrt{R}}\frac{\bar{u}(s)}{\bar{q}(s)}\bar{f}(s) &= \mathcal{L}\left[\{h(t)\}^{3/2}\right] \\
\frac{3}{16\sqrt{R}}\bar{U}(s)\bar{f}(s) &= \mathcal{L}\left[\{h(t)\}^{3/2}\right] \\
\mathcal{L}^{-1}\left[\frac{3}{16\sqrt{R}}\bar{U}(s)\bar{f}(s) \right. &= \left.\mathcal{L}\left[\{h(t)\}^{3/2}\right]\right]
\end{aligned}$$

$$\frac{3}{16\sqrt{R}}\int_0^t U(t-\zeta)F(\zeta)d\zeta = [h(t)]^{3/2} \tag{S19}$$

Equation S19 allows for the straightforward definition of the retardance $U(t)$ according to an appropriate material model. The convolution integral replaces the multiplication of $\bar{U}(s)$ and $\bar{f}(s)$ in the Laplace domain during translation back to the time domain. As mentioned previously, the approach outlined here is preferred for the AFM as it both removes the requirement of a step function in stress and measurement of the force application rate. Since an AFM-SFS experiment provides force and indentation (deformation), the data streams can be utilized directly without requiring a discrete derivative of force in time. Taking a discrete derivative can introduce undesirable oscillatory errors into the resulting dataset, and thus obscure some of the information contained within. Equation S19 represents the strain (or deformation) response of a material to the unit stress impulse.

References

1. Lee, E. H.; Radok, J. R. M. *J. Appl. Mech.* **1960**, *27*, 438–444. doi:10.1115/1.3644020.

2. Mezger, T. G. *The Rheology Handbook: For Users of Rotational and Oscillatory Rheometers*; Coatings compendia; Vincentz Network, 2006.

3. Eringen, A. C. *Mechanics of Continua*; Robert E. Krieger Publishing Co.: Huntington, NY, USA, 1980; pp 15–17.

4. López-Guerra, E. A.; Eslami, B.; Solares, S. D. *J. Polym. Sci., Part B: Polym. Phys.* **2017**, *55*, 804–813. doi:10.1002/polb.24327.
